# Case report: HLA-B35-associated optic neuritis

**DOI:** 10.3389/fopht.2024.1483937

**Published:** 2024-11-21

**Authors:** Florian H. Guillot, Andrew R. Carey

**Affiliations:** Division of Neuro-Ophthalmology, Wilmer Eye Institute, Johns Hopkins University School of Medicine, Baltimore, MD, United States

**Keywords:** human leukocyte antigen, HLA-B35, optic neuritis, optic neuropathy, Behçets disease

## Abstract

**Purpose:**

To describe a unique presentation of optic neuritis associated with positive HLA-B35.

**Observations:**

A woman presented with unilateral retro-orbital pain, mildly decreased vision, and optic disc edema with new-onset aphthous ulcers. Color vision was preserved, and no visual field deficits were noted. Diagnostic imaging demonstrated retrobulbar optic nerve enhancement, with genetic testing revealing HLA-B35 positivity. Treatment with high-dose oral steroids for 3 days resolved all symptoms, and the patient remained stable for at least 2 months.

**Conclusions and importance:**

In addition to rheumatic conditions and oral lesions, HLA-B35 can be linked to optic neuritis. This relationship highlights the need to further explore genetic risk factors associated with optic neuritis and the potential need for human leukocyte antigen (HLA) testing in unusual cases of optic neuritis.

## Introduction

Genes in the human leukocyte antigen (HLA) system are linked to numerous inflammatory, autoimmune, and malignant disorders (1). The HLA system presents processed peptides for T-cell receptor recognition, and small variations can trigger severe immune responses (1). HLA serotypes are implicated in the pathogenesis of many inflammatory eye conditions (2), including birdshot chorioretinopathy (HLA-A29), Behçet’s disease (HLA-B51), or anterior uveitis (HLA-B27) (2).

Optic neuritis (ON) encompasses a range of inflammatory optic neuropathies. Most commonly, it refers to acute inflammation or demyelination of the optic nerve characterized by vision loss, dyschromatopsia, and periocular pain. While commonly associated with multiple sclerosis (MS), ON can occur in isolation or as part of other autoimmune syndromes. Associations of non-MS ON and MS ON with various HLA loci have been identified in the literature (3–5). However, its association with HLA-B35 has only been reported in one study examining the relationships between HLA, ON, and MS in the Iranian population (5). No details on these cases have been provided. We hereby present a case of HLA-B35-associated ON.

## Case presentation

A woman in her third decade of life presented with 1 month of blurred vision and right retro-orbital pain exacerbated by eye movements. She also reported new aphthous ulcers for the last week. Her past ocular history was significant for high myopia and two remote episodes of ocular pain and decreased vision, concerning for sequential ON. The first episode occurred 12 years prior and involved bilateral eye pain with possible vision decline, which was diagnosed as dry eyes and improved after a few months on topical cyclosporine. The second episode was 8 years prior to presentation, with left-sided ocular pain and visual acuity decline to a nadir of 20/50. MRI of the brain and orbits with and without contrast performed 1 month after the onset of the second episode was negative. Symptoms resolved over a few weeks without treatment.

**Figure 1:**
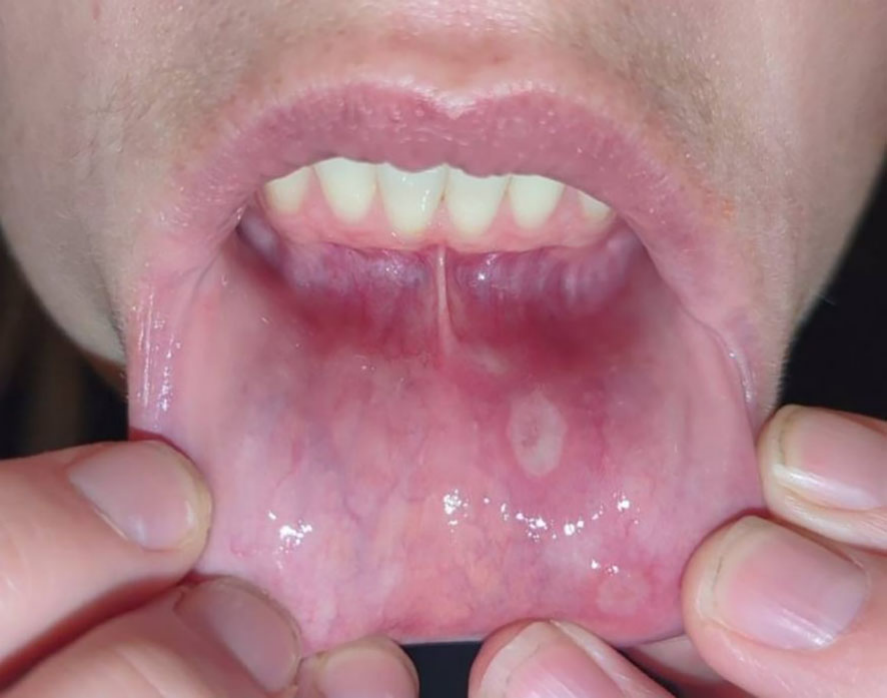
Presence of large, shallow, and white ulcers lining the lower lip mucosa of a patient presenting with HLA-B35-associated optic neuritis.

**Figure 2:**
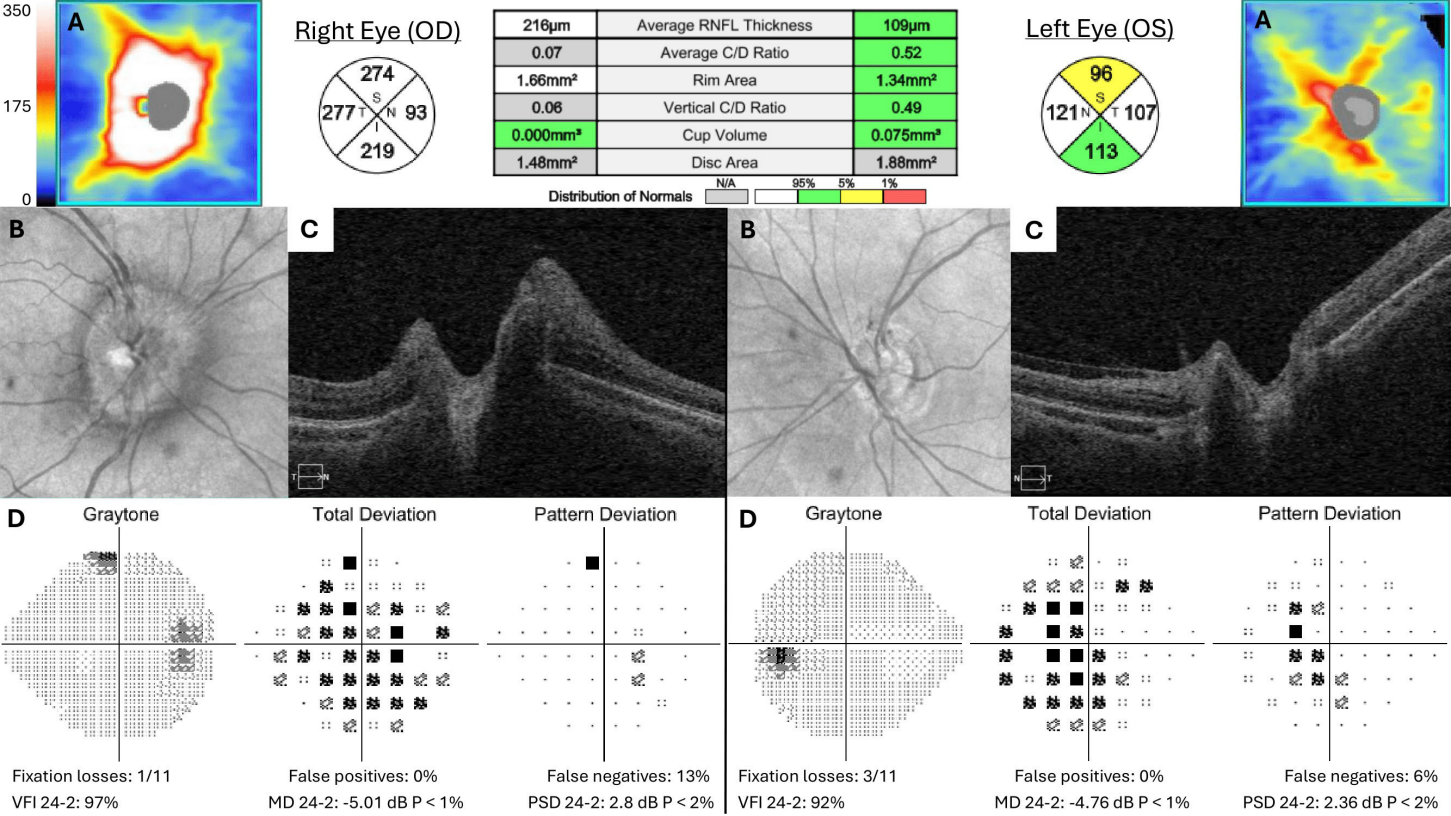
Acute presentation during third episode of optic neuritis of optic nerve head optical coherence tomography (OCT) **(A–C)** and Humphrey visual field **(D)**. Retinal nerve fiber layer (RNFL) thickness map and quadrants in microns.

On examination, visual acuity was 20/25 in the right eye and 20/30 in the left, with a 0.3 log unit relative afferent pupillary defect in the right eye. Tonometry, extraocular movements, and color vision were normal. She had a mild sensory deficit in the maxillary division of the right trigeminal nerve and aphthous ulcers on her inferior inner lip (**Figure 1**). Anterior ocular examination was unremarkable, but a dilated fundus exam showed diffuse right disc edema and trace left disc pallor. Automated visual field testing showed mild generalized depression on total deviation, but pattern deviation in the affected eye was near normal; the fellow eye showed a mild paracentral depression felt to be related to her high myopia or prior optic neuritis, and both eyes were stable prior to testing. Optical coherence tomography (OCT) was normal in the left eye but showed diffuse retinal nerve fiber layer (RNFL) thickening in the right eye (**Figure 2**).

**Figure 3:**
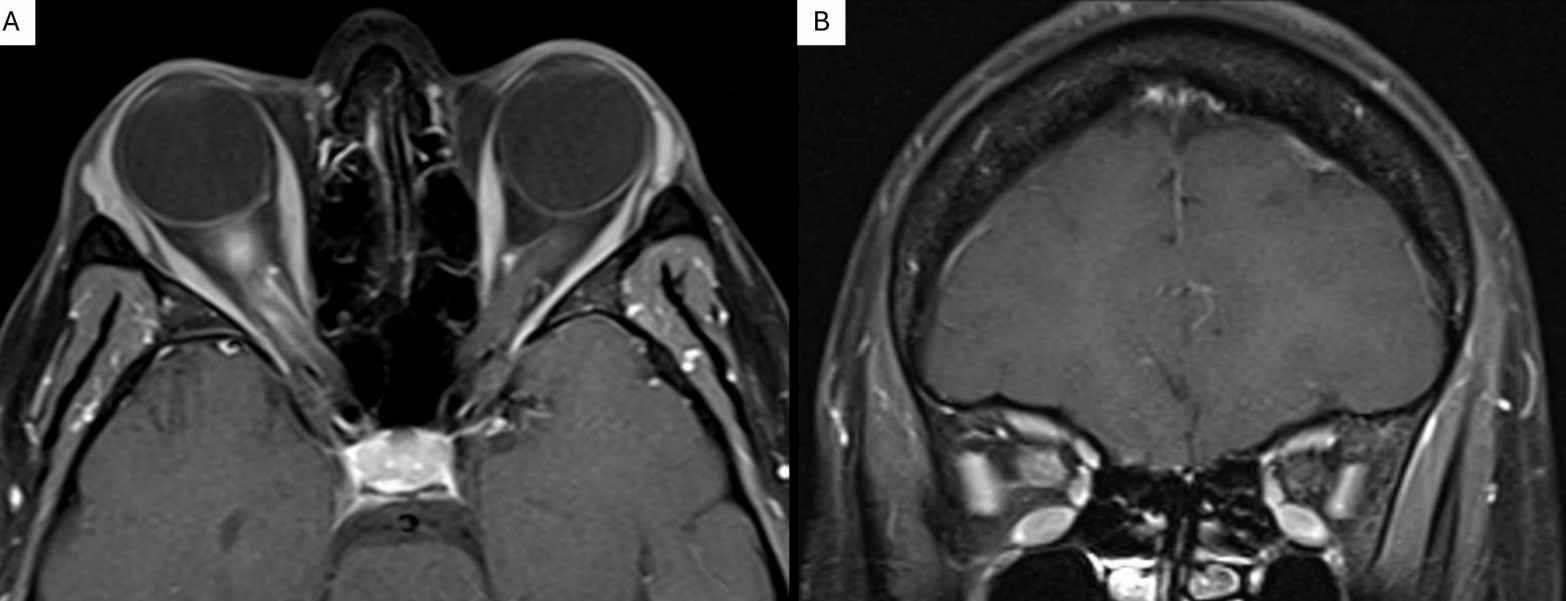
Axial **(A)** and coronal **(B)** gadolinium-enhanced MRI of a patient with HLA-B35-associated optic neuritis revealing right anterior optic nerve enhancement.

An MRI of the brain and orbits with and without contrast demonstrated right anterior optic nerve enhancement and posterior optic nerve sheath enhancement without white matter lesions (**Figure 3**). Extensive laboratory workup for infectious and inflammatory etiologies was normal, including serum aquaporin-4 antibodies, myelin oligodendrocyte glycoprotein antibodies, anti-nuclear antibodies, cytoplasmic neutrophil antibodies, *Bartonella henselae*, *Borrelia burgdorferi*, and human immunodeficiency virus. Prompted by the presence of mouth ulcers, genetic testing via next-generation sequencing was performed. Results showed the absence of the HLA-B51 allele but the presence of HLA-B7 (B*07:02:01G) and HLA-B35 (B*35:08:01G) alleles.

**Figure 4:**
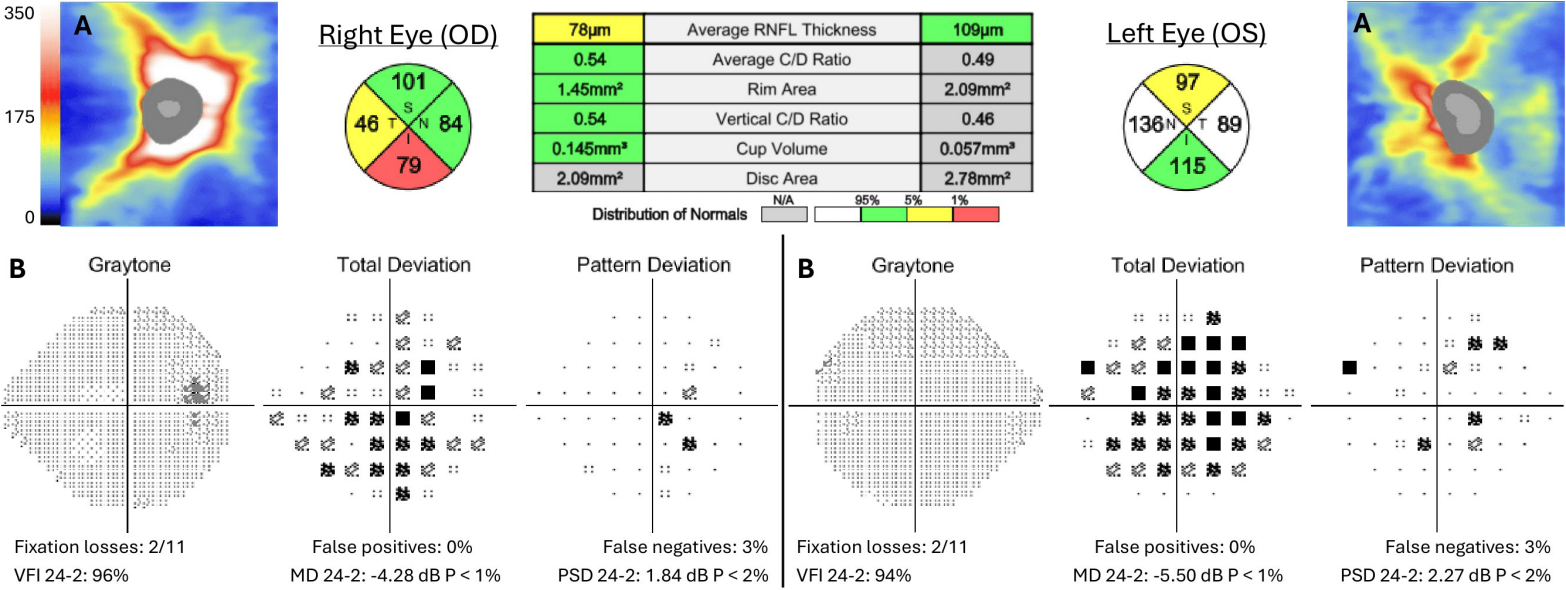
Follow-up 2 months after presentation following treatment with pulse-dose corticosteroids of optic nerve head optical coherence tomography (OCT) **(A)** and Humphrey visual field **(B)**. Retinal nerve fiber layer (RNFL) thickness map and quadrants in microns.

The patient elected to undergo treatment with the oral equivalent of pulse-dose corticosteroids (1,250 mg prednisone) for 3 days due to prolonged symptoms and pain. Oral route was chosen for practicality and equivalent efficacy compared to IV methylprednisolone (6). Five days post-treatment, visual acuity improved to 20/20 in the right eye and 20/30 in the left, with reduced retro-orbital pain and trace nasal edema of the right optic disc. OCT demonstrated improved diffuse RNFL thickening. At 1 month, pain and disc edema resolved. Vision, visual fields, and OCT measurements remained stable after 2 months (**Figure 4**). The patient later reported morning stiffness in her back and swollen knees and hands, in addition to recurrent aphthous ulcers. She was referred for evaluation by rheumatology and neuro-immunology.

## Discussion

Several unusual features stand out in this patient’s presentation. The prolonged duration of symptoms, relatively preserved visual acuity, and preserved visual fields and color vision are uncommon findings in ON. However, MRI confirmed active inflammation of the right optic nerve. The presence of disc edema and history of sequential ON suggested a possible autoimmune etiology such as Myelin oligodendrocyte glycoprotein (MOG)-antibody disease. History of oral lesions also made Behçet’s disease high in the differential diagnosis. However, antibody testing and HLA-B51 sequencing were both negative. Although a negative HLA-B51 does not preclude Behçet’s disease, this patient also lacked additional skin lesions, involvement of other organ systems, or additional signs of ocular inflammation. Isolated optic neuropathy has occasionally been reported in Behçet’s disease but remains rare (7). Infectious etiologies were deemed unlikely given the workup described above and the prompt resolution of symptoms with high-dose steroids. Multiple sclerosis was felt to be unlikely given the presence of optic disc edema, good color vision, 12-year course with lack of any brain lesions on MRI, and lack of any clinical attacks outside the optic nerves. Neuromyelitis optica spectrum disorder was felt to be unlikely given the excellent vision despite multiple clinical attacks and negative antibody testing.

Genetic testing identified the presence of HLA-B35, a finding associated with ON in only one prior study (5). This association warrants further exploration, as the pathophysiology of ON and its risk factors remain poorly understood. HLA-B35 has been implicated in various rheumatic conditions. In the Brazilian population, it was associated with recurrent minor aphthous stomatitis, a common oral mucosa disorder characterized by recurring painful mouth ulcers (8). It has also been associated with mucocutaneous lesions (9, 10), nephritis (10), and dermatitis (11) following gold treatment for rheumatoid arthritis. Lastly, HLA-B35 has also been identified as a risk factor for the development of sacroiliitis and axial spondyloarthritis (12).

Despite these links to various autoimmune and inflammatory conditions, HLA-B35 has rarely been linked to ON. In fact, a 4-year study evaluating HLA serotypes in Iranian patients found that HLA-B35 was significantly decreased in ON patients compared to controls (OR, 0.31; CI, 0.15–0.66) (5). Correlations between these two entities thus remain difficult, and additional evidence is required to evaluate its clinical significance. The association of HLA-B35 with ON in this case suggests a potential overlap between systemic autoimmune predispositions and localized inflammatory responses in the optic nerve. This aligns well with evidence from animal models of MS, where major histocompatibility complex (MHC) genes are thought to influence primarily penetrance, whereas other loci modulate specific phenotypes such as location in the brain or spinal cord, demyelination, and severity of inflammation (13).

## Conclusions

Various HLA-A and HLA-B serotypes have been found to be protective versus indicative of ON, and it remains unclear whether HLA-B35 positivity in this patient contributed to the development of ON. Overall, these results suggest that genetic factors influence ON expression and underscore the importance of considering HLA testing in the differential diagnosis of unusual presentations of ON without clear etiologies. Further research is needed to elucidate the prevalence of HLA-B35 in ON and the mechanisms by which it may contribute to ON to eventually explore its use as a potential biomarker for identifying at-risk individuals and guiding personalized treatment strategies.

## Data Availability

The original contributions presented in the study are included in the article/supplementary material. Further inquiries can be directed to the corresponding author.
